# Translation and validation of Sinhala version of modified EORTC QLQ-OH15 in oral cancer patients who receive radiotherapy with or without chemotherapy in Sri Lanka

**DOI:** 10.1186/s12903-022-02392-y

**Published:** 2022-08-19

**Authors:** Shamini Kosgallana, Prasanna Jayasekara, Prasad Abeysinghe, Marianne Hjermstad, Ratilal Lalloo

**Affiliations:** 1 Ministry of Health, Colombo, Sri Lanka; 2 Research and Surveillance Unit, Institute of Oral Health Maharagama, Maharagama, Sri Lanka; 3 Apeksha Hospital Maharagama, Maharagama, Sri Lanka; 4 Regional Advisory Unit for Palliative Care, Department of Oncology, Oslo University Hospital, Oslo, Norway; 5 European Palliative Care Research Centre, Department of Oncology, Oslo University Hospital, and Institute of Clinical Medicine, University of Oslo, Oslo, Norway; 6 School of Dentistry, University of Queensland, Brisbane, QLD 4006 Australia

**Keywords:** Oral cancer, Oral health related quality of life, Radiotherapy, EORTC QLQ-OH15, Factor analysis, Reliability, Responsiveness

## Abstract

**Background:**

The recognition of patient-reported outcomes for oral cancer is important in improving patients’ quality of life. The aim of this study was to translate and validate the modified Sinhala version of the European Organization for Research and Treatment of Cancer Quality of Life Questionnaire Oral Health Module (EORTC QLQ-OH15).

**Methods:**

A cross-sectional study was conducted to validate the EORTC QLQ-OH15 that was modified after adding two questions to the original questionnaire. The two questions added were ‘difficulty in opening the mouth wide’ and ‘trouble with talking’ which affect oral health related quality of life (OHRQOL) of oral cancer patients receiving radiotherapy. The Sinhala translated modified EORTC QLQ-OH15 and already validated the core questionnaire EORTC QLQ-C30 were self-completed by 85 adult oral cancer patients who received initial anti-cancer treatment with radiotherapy with or without chemotherapy. Content and face validity were examined by an expert panel. Construct validity was confirmed by using factor analysis, multi-trait scaling analysis, and known group comparison. Reliability was assessed by internal consistency, test–retest reliability by Wilcoxon Signed Ranks Test and intra class correlation coefficient. Responsiveness to change was assessed.

**Results:**

The majority of participants (58%) were aged 50–69 years and 84% were males. Nearly 32% had cancer of the anterior two thirds of the tongue. Of the sample, 66% received chemo radiotherapy. Thirteen items were included for the factor analysis. They were loaded for four factors. Three scales *‘Eating problem’*, ‘*Gum and Speech problem’* and ‘*Soreness*’ loaded with 5, 4 and 3 items respectively and single item *‘teeth’* to a one factor with the total variance explained was 72.74%. Mann–Whitney U tests for all three scales were statistically significant confirming the ability of the modified EORTC QLQ-OH15 to detect expected differences in OHRQOL in clinically different groups. Cronbach’s alpha for all the scales were more than 0.8. Wilcoxon Matched Paired Sign Rank Test showed highly significant results (*p* < 0.05) for all three scales revealing high responsiveness.

**Conclusions:**

The modified Sinhala version of the EORTC QLQ-OH15 is a valid, reliable tool that can be used to measure OHRQOL in oral cancer patients who receive radiotherapy with or without chemotherapy.

## Introduction

In the last decades, several validated quality of life tools have become available to measure health related quality of life (HRQOL) that can be used for all types of cancer [1]. The World Health Organization has identified oral health as an important issue in relation to health related quality of life (HRQOL) [2–5]. Researchers have reported three categories of oral health related quality of life (OHRQOL) measurements. They are social indicators, global self-ratings and multiple items questionnaires. Social indicators are used to assess the consequences of oral conditions at the community level. Global self-ratings of OHRQOL are single-item ratings, which provide information about their own oral health, whereas multiple item questionnaires are the most commonly used method to assess OHRQOL [5].

OHRQOL instruments have been developed as general, i.e. for use in population wide studies or surveys as well as specific tool for defined diseases and/or population categories [6, 7]. Disease-specific measures are developed to measure disease related symptoms that may impact on oral health or how oral health symptoms and problems may impact overall quality of life and the patient’s condition [8]. Oral health problems pertain to a number of different diseases and treatments, within and outside the oral cavity. This applies to oral cancer in particular. There is growing interest in understanding the impact of the oral cancer and its treatment on the OHRQOL of cancer survivors and the need of cancer-specific tools for the assessment of OHRQOL. The European Organization for Research and Treatment of Cancer (EORTC) Quality of Life Group has developed a quality-of-life questionnaire oral health module (EORTC QLQ–OH15) to assess OHRQOL in patients with any type of cancer. The reliability, validity, and psychometric properties of the EORTC QLQ-OH15 questionnaire was first published in 2016 after being field tested in an international heterogeneous sample of cancer patients [9, 10]. The original EORTC QLQ-OH15 is a self-administered questionnaire. It comprises eight item scale named OH-QOL, 3 single items (sticky saliva/ mouth soreness/sensitivity to food/drink) and 2 dichotomous items (Yes/No) regarding information received and use of dentures. These are followed by two items that are to be completed contingent on the response being YES for the previous two. The other 13 items are rated on a four-point Likert scale (‘not at all’, ‘a little’, ‘quite a bit’ and ‘very much’) [9]. This was developed as a supplementary module to be used with the EORTC quality-of-life core questionnaire (EORTC QLQ-C30) that has already been translated into Sinhala and validated in Sri Lanka. This also applies to another EORTC tool, the specific head and neck EORTC QLQ-H&N35 module [11].

Even though the number one cancer in males in Sri Lanka is oral and pharyngeal cancers [12] with an age standardized incident rate of 19.1 per 100,000 male population in 2019 [13], there are no data published on OHRQOL of oral cancer patients. Even though the treatments are meant to be improving the patients’ OHRQOL, they give array of side effects and complication which will deteriorate OHRQOL of these oral cancer patients [14, 15]. The most common treatment modality for oral cancer is radiotherapy (RT) which is given post-surgically or alone or in combination with chemotherapy in Sri Lanka [12, 13]. Even with the vast knowledge of the occurrence of these oral complications due to RT, research conducted to measure OHRQOL is limited.

OHRQOL reveals patient centered outcomes, changes of OHRQOL due to treatments by assessing pre-treatment and post-treatment outcomes. Evidence from such research can assist with developing treatment protocols with minimal negative consequences [3]. Therefore, it is a necessity of a tool to evaluate the quality-of-life of such patients to take necessary initiatives to enhance the quality-of-life. It is utmost important therefore, to translate and validate a tool to assess OHRQOL for the Sri Lankan context.

The aim of this study was to translate the EORTC QLQ-OH15 into Sinhala language and assess the validity, reliability, responsiveness and acceptability of modified EORTC QLQ-OH15 in oral cancer patients who receive RT with or without chemotherapy in Sri Lanka.

## Methods

### Study design and duration

The study consisted of two components. First, the modified questionnaire was translated into Sinhala language and adapted to Sri Lankan culture. Secondly, the Sinhala version was validated for psychometric properties. The study was carried out during 2015 and 2016.

### Modification, translation and pilot testing of the modified EORTC QLQ- OH15

A Modified Delphi Technique was carried out among eleven consultants in oncology and six medical officers (doctors) at the Apeksha hospital, the main tertiary cancer care hospital in Colombo, Sri Lanka. This method was used to assess the adequacy of the questions in the questionnaire to capture all the relevant oral side effects of RT treatment for oral cancer patients. The oncologists and medical officers were asked to propose relevant important questions for this patient group that they thought were missing. Based on their feedback, two questions were added, namely ‘difficulty in opening the mouth wide’ and ‘trouble with talking’. These additional new questions were found in EORTC questionnaires which were already translated to Sinhala and validated (EORTC disease specific quality of life modules for head and neck and, eosophageal cancer) [10]. This 17-item version was then circulated among all the participants of Modified Delphi Technique and their consensus was confirmed. Thus, the modified EORTC QLQ-OH15 questionnaire in Sinhala comprised of 17 questions.

Then, the 15 items in the original EORTC QLQ-OH15 were translated into Sinhala language guiding the translation procedure of EORTC Quality of Life Group [16]. The translation of the English version to Sinhala (forward translation) was carried out independently by two translators. Both were native speakers of Sinhala language with a high level of fluency in English. The first and the second translations were merged into one single provisional forward translation by the principal investigator. The problems identified were discussed with the translators, and a reconciled version of the translations was produced.

Backward translation (reconciled Sinhala version into English) was performed by a different two translators who were native English speakers with a high level of fluency in Sinhala language. Translations were done independently by the translators without the knowledge of the original English version. The English translations were compared by the principal investigator with the original questionnaire following a workflow that was consistent with the reconciliation process described under reconciliation of the two forward translations by EORTC Quality of Life Group [17]. The simplest and commonly used words in day-to-day life of Sinhalese people were included.

The translated questionnaire was pilot-tested among 12 oral cancer patients who receive RT with or without chemotherapy and structured interviews were conducted with each patient individually. These patients were not included in the main validation study. The structured interviews focused on each item separately to determine whether the wording used in any of the translated items was: (a) Difficult to answer, (b) Confusing, (c) Difficult to understand, (d) Upsetting/offensive and (e) Whether the patient would have asked the question in a different way. Based on the interviews, modifications were made to the provisional version. Translation report consisting of the translation process, interim forward and backward translations and report on pilot testing were submitted to the EORTC quality of life group to review. The final Sinhala translation of EORTC QLQ-OH15 was approved by EORTC Quality of Life Group. The modified EORTC QLQ-OH15 with two additional questions was used for the validation.

### Validation of the modified EORTC QLQ-OH15

**Fig. 1:**
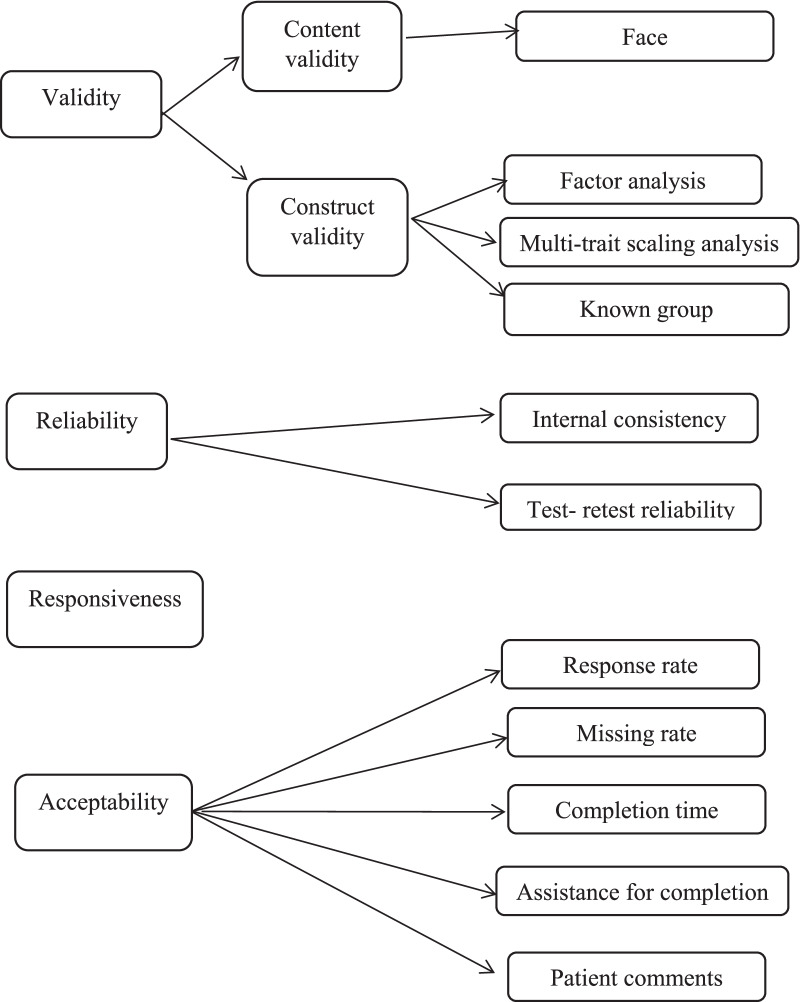
Schematic representation of methods used to assess validity, reliability, responsiveness and acceptability

The modified EORTC QLQ-OH with 17 items (Sinhala version) was tested for content validity, face validity, construct validity, the reliability, responsiveness and acceptability (Fig. 1).

A multidisciplinary expert panel consisted of two Consultants in Community Dentistry, one Consultant in Maxillofacial Surgery, five Consultants in Oncology, one Registrar in Oncology and a Senior Medical Officer involved in evaluating content validity and face validity. According to the consensus-based standards for the selection of health measurement instruments (COSMIN) definitions, content validity is the adequacy of reflecting the construct that is going to measure and the face validity has considered as an aspect of content validity in patient reported outcome instruments [18]. Each item was assessed for appropriateness of the items, its relevance in assessing OHRQOL in patients with oral cancer, appropriateness of the wording used and acceptability in the local context by the expert panel.

#### To assess the psychometric properties, the questionnaire was administered among 85 patients with oral cancers

The sample size was taken to a subject to item ratio of 5:1 considering the recommendation of Tabachnick and Fidell [19].The study sample was 85 for 17 items in the questionnaire. Therefore, 85 patients who met the inclusion criteria, either waiting for RT or having completed RT were recruited from outpatient clinics and hospital wards at Apeksha hospital, Colombo, Sri Lanka. Oral cancer patients who had undergone surgery, patients who were in their terminal phase of the cancer and patients who were unable to participate in the interviews due to obvious cognitive impairment, psychological disturbance or language problems were excluded from the study. Eligibility for the study was confirmed by reviewing their medical records.

After obtaining informed written consent, and having received thorough information about the study, patients completed the Sinhala versions of the modified EORTC QLQ-OH15 and previously validated EORTC QLQ-C30 questionnaires by themselves. Assistance was provided as required. Socio-demographic data of the participants was gathered using a pre-tested interviewer administered questionnaire. Clinical information was obtained from clinic and hospital records. After two weeks of 1st administration, 25 patients who did not receive any medical interventions affecting oral status after the first assessment were recruited and asked to complete the modified EORTC QLQ-OH15 questionnaire for the second time (to assess test–retest reliability). Further, another 25 patients whose condition was expected to change due to RT were recruited on the 1st or 2nd day of the RT treatment and filled the questionnaire again after two weeks of 1st administration (to test the responsiveness to change). Ethical approval for the study was obtained from the Ethics Committee, Faculty of Medicine, University of Colombo. Sri Lanka. The approval number is EC-15-200.

### Statistical analysis

The data were analyzed using Statistical Package of Social Sciences version 22. Principal component analysis (PCA) was performed to identify underlying variables, factors that explain the pattern of correlations within the set of observed variables to assess construct validity. Data screening was performed to assess missing data and the factorability. Assessment of the suitability of the data to carry out factor analysis was performed. Kaiser–Meyer–Olkin Measure of Sampling Adequacy should be more than 0.50 and Bartlett's test of Sphericity should be significant (*p* < 0.05) to consider the suitability for factor analysis [20–23]. Normality was assessed by using histograms and tests of normality; Kolmogorov–Smirnov and Shapiro–Wilk tests. Valid factor loading values cannot be produced if the variables are highly correlated (it should be > 0.00001). Therefore, the dataset was tested for multicollinearity [20]. The values of correlation matrix above 0.3 was suggested as another mode of checking the suitability of carrying out the factor analysis [20, 23].

### Selection of variables

The two ‘Yes’ or ‘No’ answer questions; ‘Have you worn dentures?’ and ‘Have you received any information about possible dental or mouth problems?’ were not included in the analysis. Only one patient had worn dentures during the last week and only 11 had answered ‘Yes’ for the question ‘Have you received any information about possible dental or mouth problems?’ and therefore only 11 had answered the question ‘Have you been satisfied with the amount of information you received about possible dental or mouth problems?’ Finally, 13 variables were included for the factor analysis [22].

### Principal component analysis

Initially PCA was performed excluding the additional new questions (mouth opening and speech). The item loading to factors were not similar to the analysis by the EORTC group even without adding the two new variables [9]. Then PCA was performed after adding two new variables. The correlation matrix between components one and two had a correlation of 0.367 which was more than 0.32. This indicates the choice of rotation method was oblimin compared to varimax [24].

Multi-trait scaling analysis which assesses convergent validity and discriminant validity was performed using Spearman’s test. When correlation coefficients of the items in its own scales are more than 0.4, the tool is considered having a good convergent validity. Good discriminant validity was achieved when each item has a lower correlation with other scales than that of their own scale [25, 26]. Known group comparison was performed between the groups of patients before RT and after RT as other methods of assessing construct validity. The median scores of *‘Eating problem’*, ‘*Gum and Speech problem’* and ‘*Soreness*’ scales were compared by known group comparison, to assess whether the modified EORTC QLQ-OH15 was able to detect expected differences in OHRQOL of these clinically different groups [23]. Correlation between the Modified EORTC QLQ-OH15 and EORTC QLQ-C30 scales were assessed using Spearman’s correlation test.

Reliability was assessed using two methods; internal consistency by Cronbach’s Alpha and test re-test reliability by Wilcoxon Signed Ranks Test and Interclass correlation coefficient (ICC). Correlations at least 0.7 are considered acceptable and 0.8 and above are considered having a good reliability [10]. Responsiveness was tested by Wilcoxon Matched Paired Sign Rank Test. It permits to assess the suitability of the scales and items to detect small changes that are clinically important [25].

## Results

Eighty-five oral cancer patients filled the 17–item questionnaire and 63.5% of them were able to complete the questionnaire without assistance. The response rate was 100% and there were no missing data. It took less than 5 min to complete the questionnaire. All the participants were above 30 years old, and majority (57.6%) were in 50–69 age group. Of the sample 83.5% were males and 94.1% were married. The monthly income was less than fifteen thousand Sri Lankan Rupees for 50.6% of the patients. The anterior two-thirds of the tongue (31.8%) and the buccal mucosa (29.4%) were the most common sites of the primary tumor and 75.3% were in their late stage (Stage III and IV). Chemo-RT was recommended as the treatment modality in 65.9% of the patients. The questionnaire was completed by 70.6% of patients prior to RT and 29.4% of patients after RT (Table 1).

**Table 1:** Distribution of the Validation study population by socio demographic and clinical characteristics (n = 85)

Characteristics	Frequency	Percentage (%)
*Age*		
30–49	22	25.9
50–69	49	57.6
> 70	14	16.5
*Sex*		
Female	14	16.5
Male	71	83.5
*Civil status*		
Married	80	94.1
Unmarried	5	5.9
*Education level*		
Up to grade 5	19	22.4
Up to GCE O/L	53	62.3
Up to GCE A/L	11	12.9
Diploma/Degree	2	2.4
*Employment status*		
Unemployed	16	18.8
Self employed	55	64.7
Employed	12	14.1
Pensioner	2	2.4
Income*		
LKR < 15,000/=	43	50.6
LKR 15,000/= − 30,000/=	28	32.9
LKR > 30,000/=	14	16.5
*Site of the cancer*		
Lip	8	9.4
Anterior two-thirds of the tongue	27	31.8
Buccal mucosa	25	29.4
Floor of the mouth	9	10.6
Hard palate	4	4.7
Lower and upper alveolar ridge	2	2.4
Retromolar trigone	10	11.8
*Stage*		
Early stage (stage I and II)	21	24.7
Late stage (stage III and IV)	64	75.3
*Treatment modality*		
Radiotherapy	29	34.1
Chemo-radiotherapy	56	65.9
*Treatment status*		
Before treatment	60	70.6
Post treatment	25	29.4

*1US$ = 198LKR

Kaiser–Meyer–Olkin Measure of Sampling Adequacy obtained in this study was 0.77, hence the sample size taken for the validation was adequate. Bartlett's Test of Sphericity was statistically significant (*p* < 0.0001) supporting the factorability of the correlation matrix. Histograms showed skewed distributions and tests of normality; Kolmogorov–Smirnov and Shapiro–Wilk tests gave statistically significant results (*p* < 0.0001) indicating distributions were not normal. Correlation was 0.001 for this dataset indicating that there was no multicollinearity. When considering the correlation matrix most of the values were more than 0.3 revealing the suitability of carrying out factor analysis.

When PCA was performed, the results showed four factors exceeding Eigenvalue more than one and the total variance explained was 72.74%. When observing the component matrix, the variables had loaded to four factors with large coefficients (suppressed by 0.5 coefficients). Five items loaded strongly into the first factor (Table 2) and was named the “*Eating problem*” scale as all of them were related to problems when eating. Four items loaded into the second factor was named as “*Gum and Speech problem*” scale and three items loaded to the third factor was named as “*Soreness*” scale. Problem with teeth item was analyzed as a single item named as “*Teeth”*. The scores were converted to a 0–100 scale by linear transformation suggested for symptom scales and items by the EORTC group. Higher scores represent more symptoms or problems [27].

**Table 2:** Pattern matrix of four factor solution

Items	Component			
	1	2	3	4
Have you had pain in your gums?		0.941		
Have you had problems with bleeding gums?		0.809		
Have you had lip sores?			0.696	
Have you had problems with your teeth?				0.862
Have you had soreness in your mouth?			0.515	
Have you had sores in the corners of your mouth?			0.888	
Have you had a dry mouth?	0.911			
Have you had sticky saliva?	0.887			
Has your mouth been sensitive to food and drink?	0.626	0.411		
Have food and drink tasted different than usual?	0.866			
Have you had problems eating solid foods?	0.722			
Have you had difficulty in opening your mouth wide?		0.497		
Have you had trouble with talking?	0.460	0.456		

Extraction Method: Principal Component Analysis

Rotation Method: Oblimin with Kaiser Normalization

Rotation converged in 12 iterations

Item convergent validity was confirmed for all the items in all the scales and the values were above 0.4. The correlation coefficients of all the items were lower for the other scales than the own scale confirming the discriminant validity (Table 3). Results of known group comparison revealed that Mann–Whitney U tests for all three scales were statistically significant (p < 0.0001). It shows higher symptoms (lower OHRQOL) in post RT group than pre-RT group (Table 4). *‘Eating Problem’* scale showed moderate correlation with most of the scales of EORTC QLQ-C30 and weak correlations were found with *‘Emotional functioning’, ‘Cognitive functioning’* and *‘Nausea and vomiting’.* The ‘*Gum and Speech problem’* and *‘Soreness’* scales showed weak correlation with most of the scales of EORTC QLQ-C30 (Table 5).

**Table 3:** Multi-trait scaling matrix of correlation coefficients for the study group

Item	Scales		
	Eating	Gum and speech	Soreness
Have you had a dry mouth?	0.767^**^	0.380^**^	0.108
Have you had sticky saliva?	0.839^**^	0.527^**^	0.346^**^
Has your mouth been sensitive to food and drink?	0.785^**^	0.642^**^	0.301^**^
Have food and drink tasted different than usual?	0.892^**^	0.551^**^	0.345^**^
Have you had problems eating solid foods?	0.855^**^	0.579^**^	0.478^**^
Have you had pain in your gums?	0.386^**^	0.784^**^	0.251^*^
Have you had problems with bleeding gums?	0.344^**^	0.625^**^	0.341^**^
Have you had difficulty in opening your mouth wide?	0.438^**^	0.762^**^	0.511^**^
Have you had trouble with talking?	0.386^**^	0.784^**^	0.251^*^
Have you had lip sores?	0.303^**^	0.499^**^	0.764^**^
Have you had soreness in your mouth?	0.385^**^	0.452^**^	0.791^**^
Have you had sores in the corners of your mouth?	0.177	0.199	0.676^**^

Item correlations with its own scale for item convergent validity are shown in bold typing

**Correlation is significant at the level 0.01

*Correlation is significant at the level 0.05

**Table 4:** Distribution of median scores for scales in pre RT and post RT group (known group comparison)

Scales	Median of clinical category		Significance*
	Pre RT n = 60	Post RT n = 25	
Eating problem	20.0	73.3	U = 129.00
			Z = − 6.0
			P = 0.000
Gum and Speech problem	8.3	41.7	U = 261.0
			Z = − 4.8
			P = 0.000
Soreness	11.1	33.3	U = 452.5
			Z = − 2.9
			P = 0.000

*Mann–Whitney U test

**Table 5:** Correlation between modified EORTC QLQ-OH15 and EORTC QLQ-C30 scales

EORTC QLQ-C30 scales	Modified EORTC QLQ-OH15 scales		
	Eating problem	Gum and speech problem	Soreness
Global health status	− .535	− .530	− .305
Physical functioning	− .664	− .496	− .324
Role functioning	− .496	− .558	− .368
Emotional functioning	− .283	− .431	− .442
Cognitive functioning	− .393	− .274	− .196*
Social functioning	− .566	− .544	− .341
Fatigue	.535	.519	.460
Nausea and vomiting	.348	.194*	.247
Pain	.565	.483	.356

The negative direction denotes lower QOL scores with higher symptom burden on the QLQ-OH15

*Not statistically significant. All unmarked are statistically significant (*p* < 0.05)

Cronbach’s alpha coefficients of all the scales were more than 0.8, which represent high reliability. For the test–retest reliability, ICC for all three scales were 7.0 or more (Table 6) and all three scales were non-significant for the Wilcoxon Signed Ranks Test indicating no differences in responses over time. Wilcoxon Matched Paired Sign Rank Test results showed highly significant results for all three scales revealing suitability to detect small changes that were present in patients within 14 days of RT course (Table 7). Therefore, responsiveness of the questionnaire was highly satisfactory.

**Table 6:** Reliability statistics of the modified EORTC QLQ-OH15

Scales	No. of items	Cronbach's Alpha	ICC (95% CI)
Eating problem	5	0.989	0.980 (0.948–0.992)
Gum and speech problem	4	0.978	0.946 (0.859–0.979)
Soreness	3	0.848	0.700 (0.365–0.873)

**Table 7:** Responsiveness results of Wilcoxon matched paired sign rank test

Scales	Wilcoxon matched paired sign rank test	
	Z	Significance*
Eating problem	− 4.133	0.0001
Gum and speech problem	− 3.276	0.001
Soreness	− 4.155	0.0001

*Based on negative ranks

## Discussion

The results revealed that this Modified EORTC QLQ-OH15 questionnaire was capable of detecting small changes due to treatments and a reliable valid tool to measure OHRQOL of oral cancer patients who receive RT with or without chemotherapy with three symptom scales and one symptom item.

According to the literature survey, this is the first study that validates this tool in an oral cancer population. Furthermore, it was a recently developed questionnaire, and very limited testing of this factor structure has been conducted to date. A study carried out with Iranian cancer patients using EORTC QLQ-OH17 which consisted of two more variables also showed a different factor loading than the original EORTC QLQ-OH15 [28]. Therefore, there was a need to perform factor analysis to assess the construct of the questionnaire as this modified questionnaire consisted of two more questions (difficulty in mouth opening and problems with speaking) and it was validated only in oral cancer patients treated with RT with or without chemotherapy.

When selecting variables for PCA, two variables regarding dentures and information had to be removed. Only one participant out of 85 had worn dentures during the last week. The reason could be that, wearing dentures was not considered to be a priority among oral cancer patients as the majority of participants were of a low socioeconomic status and/or found them uncomfortable with the cancer being in their mouth.

Only 11 had answered ‘Yes’ to the question ‘information they received about possible dental, or mouth problems’ and those 11 participants answered to the question regarding the satisfaction of information given. These results should not be neglected though these questions were not included in PCA. The health staff should be more concern in providing relevant and accurate information to their patients about the consequences of RT and oral care [29].

Results of PCA revealed that the items were loaded to three-factor model and did not overlap with the construct of the original tool even without adding two new variables. Therefore, rotation with added items was performed and result revealed a four-factor model. Dry mouth, sticky saliva, sensitivity to food and drink, food and drink tasting different than usual and problems when eating solid foods had loaded to ‘factor one’ and was justifiable from a clinical scenario as all these symptoms are related to problems when eating (*‘Eating problem’* scale).

*‘Problem with talking’* has loaded to both ‘factor one’ and ‘factor two’. After considering the clinical relationship with the items loaded and expert opinion, it was decided to retain *Problem with talking’* with ‘factor two’. This decision was most probably correct according to the Bachman (1990). He stated, *"In the exploratory mode, we attempt to identify the abilities, or traits that influence performance on tests by examining the correlations among a set of measures"* [30]. Lip sores, soreness in mouth, sores in the corners of the mouth loaded to one factor (‘*Soreness*’ scale) and this may be due to the fact that people perceive soreness in similar manner even with the varied sites. The variable loading has fulfilled the saying that at least three variables should load to one factor for statistical recognition [22].

Multi-trait scaling analysis confirmed the item convergent validity and item discriminant validity for all the items. The item convergent validity and discriminant validity confirmed that the decision taken to retain ‘*Problems with speaking’* with ‘factor two’ was correct. Known group comparison results indicated the scores for pre-treatment and post-treatment RT groups were statistically significant for all three scales. These results confirmed that the factor loadings were precise, and they have an ability to capture the differences in dissimilar situations. Correlation between modified EORTC QLQ-OH15 scales and scales of EORTC QLQ-C30 were mainly weak. Strong correlations were not found. This revealed that additional important information about relevant quality of life issues in patients with oral cancers can be gathered by using the modified EORTC QLQ-OH15 supplementary to EORTC QLQ-C30.

When considering the reliability of the modified EORTC QLQ-OH15, Cronbach’s alpha was more than 0.8 for all three scales whereas the 0.55 for oral health scale in the original questionnaire used in Chinese cancer patients [31]. Test–retest reliability were highly satisfactory in all three scales whereas intra class correlation coefficient revealed the same except *‘Soreness’* scale showing moderate reliability.

The responsiveness had been measured in a research conducted in China considering the head and neck cancer patients in their sample between the pretreatment scores and scores of 2–3 days post treatments. They found statistically significant differences only in the OHQOL scale and the sticky saliva item. Sensitivity and sore mouth items had not shown significant differences [31]. The responsiveness for all three scales in the current study was highly significant whereas the original questionnaire validation had not shown significant results.

The possible reasons for findings of this study were inclusion of only the oral cancer patients and the patients who receive only RT with or without chemotherapy. The results may had been different if the oral cancer patients who have undergone surgery were included. The validation of this EORTC QLQ-OH 15 was done using all types of cancer patients [9, 28, 31].

This tool can be used to measure OHRQOL of oral cancer patients during any time of their treatment course and in oral cancer survivors. Further, this can be used in head and neck cancer patients whose treatment of choice is RT with or without chemotherapy as the symptoms related to OHRQOL are similar. The modified EORTC QLQ-OH15 is useful in clinical trials as it showed highly significant results for responsiveness. When this is used with EORTC QLQ-C30, extra knowledge can be gathered related to quality-of-life of oral cancer patients. Known group comparison results confirmed that the factor loadings were precise, and they have an ability to capture the differences in dissimilar situations.

The strength of the study is that the factor loading in PCA was confirmed by the results of multi -trait scaling analysis, known group comparison and correlation to the scales in EORTC QLQ-C30. Reliability was assessed in several ways and responsiveness to small clinical changes was also confirmed in this Modified EORTC QLQ-OH15. The limitations of the study were that the validation was performed only in the main tertiary cancer care hospital in Sri Lanka and only in oral cancer patients treated with RT with or without chemotherapy. Validating the tool in different settings and in oral cancer patients undergoing other treatment modalities are recommended.

## Conclusion

The modified EORTC QLQ-OH15 had shown a very good responsiveness to change, and this tool is a valid, reliable tool that can be used to measure OHRQOL in oral cancer patients who receive RT with or without chemotherapy.

## Data Availability

The datasets used and/or analyzed during the current study are available from the corresponding author on reasonable request.

## Abbreviations

EORTC QLQ-OH15: European Organization for Research and Treatment of Cancer Quality of Life Questionnaire Oral Health Module
OHRQOL: Oral health related quality of life
HRQOL: Health related quality of life
EORTC: European Organization for Research and Treatment of Cancer
EORTC QLQ-C30: European Organization for Research and Treatment of Cancer quality of life core questionnaire
RT: Radiotherapy
PCA: Principal component analysis
ICC: Interclass correlation coefficient
